# Protective Coatings Based on the Organosilicon Derivatives of Fatty Acids Obtained by the Thiol-Ene Click Reaction

**DOI:** 10.3390/ma17174432

**Published:** 2024-09-09

**Authors:** Karol Szubert, Albert Liberski

**Affiliations:** 1 Faculty of Chemistry, Adam Mickiewicz University, Uniwersytetu Poznańskiego 8, 61-614 Poznan, Poland; 2 Construction Research and Innovation Laboratory, Innova Seminars, Doha 24144, Qatar; chemikszkot@gmail.com

**Keywords:** fatty acids, sol-gel processes, organically modified silanes, thiol-ene click reaction, concrete, protective

## Abstract

This article describes the synthesis of a hydrophobic protective coating for concrete based on a silane derivative of fatty acids. The coating was obtained through a thiol-ene click addition reaction using methyl oleate and 3-mercaptopropyltrimethoxysilane in the presence of the photoinitiator 2,2-dimethoxy-2-phenylacetophenone (DMPA). This reaction proved to be more efficient compared with other tested (photo)initiators, considering the double bond conversion of oleate. The coating was applied to concrete using two methods: immersion and brushing. Both methods exhibited similar consumption of methyl oleate-based silane (UVMeS) at approximately 20 g/m^2^. The hydrophobic properties of the coatings were evaluated based on the contact angle, which for the modified surfaces was above 93°, indicating their hydrophobic nature. The penetration depth of the silane solution into the concrete was also studied; it was 5–7 mm for the immersion method and 3–5 mm for the brushing method. The addition of tetraethoxysilane (TEOS) to the silane solution slightly improved the barrier properties of the coating.

## 1. Introduction

Concrete plays a crucial role as a fundamental building material, forming the basis of many civilizational achievements [1]. Despite its long history, concrete continues to attract attention due to its wide range of applications and the potential benefits of improving its technology. Even seemingly minor advances in concrete technology can lead to significant economic effects on a large scale.

For example, the incorporation of supplementary cementitious materials (SCMs) such as fly ash and slag into concrete mixes improves the durability and sustainability of concrete. The use of fly ash in concrete can lead to an increase of 20–30% in the useful life of concrete structures, reducing the need for repairs and replacements [2].

One of the main durability challenges for concrete is its inherent porosity, which allows water and harmful chemicals to infiltrate. This infiltration can lead to the corrosion of embedded steel reinforcements, which is a significant concern as it causes the expansion of the steel, leading to the cracking and spalling of the surrounding concrete [3,4]. Additionally, water intrusion can promote the growth of microorganisms, which can further deteriorate the concrete over time [4]. Freeze–Thaw cycles are another critical issue; When water within the concrete freezes, it expands, causing internal pressure that can crack and damage the concrete structure [4].

These vulnerabilities highlight the importance of enhancing the resistance of concrete to water penetration to improve its longevity and structural integrity.

Traditional methods of waterproofing concrete surfaces, such as using waterproof paints, can have significant limitations. One key issue is that while these paints prevent water from entering the exterior, they also inhibit the release of moisture from within the concrete structure. This trapped moisture can negatively affect the durability of concrete, potentially leading to problems such as blistering, peeling, and reduced protective coating longevity [5,6]. Additionally, waterproofing systems that rely on surface coatings can be problematic in varying environmental conditions, where proper curing and application are crucial for ensuring effectiveness [6].

Waterproofing materials based on bituminous and polymer components have also been widely employed to protect concrete. Bituminous materials are often used due to their excellent adhesion properties, flexibility, and ability to form a continuous waterproof membrane. These materials, which include asphalt and bitumen-based emulsions, are particularly effective in preventing water ingress [7]. However, bituminous coatings can be prone to degradation under UV exposure and high temperatures, which may lead to cracking and a reduction in waterproofing efficacy over time. Additionally, the application of bituminous coatings typically involves heating and handling hazardous materials, which can present safety and environmental concerns [8].

On the other hand, polymer-based waterproofing materials, such as polyurethane and acrylic coatings, offer enhanced elasticity, chemical resistance, and durability. These materials are particularly beneficial in environments with dynamic structural movement as they can stretch and return to their original shape without cracking [9]. However, polymer-based systems can be sensitive to substrate preparation and curing conditions. Improper application can lead to defects such as pinholes or bubbles, which compromise the waterproofing effectiveness. Furthermore, some polymer systems may degrade under UV exposure unless UV stabilizers are added, increasing the complexity and cost of these systems [10].

Moreover, the tribological performance of superhydrophobic surfaces is another primary factor that determines their overall effectiveness. Research has shown that designing superhydrophobic surfaces with superior tribological properties significantly contributes to their practical application. For instance, Yang et al. demonstrated that the tribological performance of surfaces could be enhanced by adjusting surface wettability through chemical etching and modification, as seen in the treatment of AA3003 aluminum alloy [11].

In recent years, research has shifted towards advanced coating technologies like sol-gel coatings, particularly hybrid organic–inorganic sols, which offer a promising alternative to traditional waterproofing methods. The sol-gel process allows for the formation of ceramic-like coatings at relatively low temperatures, making it suitable for treating concrete surfaces. These coatings can provide a hydrophobic layer while maintaining the breathability of the concrete, preventing the build-up of moisture within the structure [12,13].

Hybrid organic–inorganic sols are of particular interest due to their ability to combine the flexibility of organic materials with the durability of inorganic components. These coatings have shown effectiveness in enhancing the water-repellent properties of concrete surfaces. For instance, research has demonstrated that organosilane-modified sols can penetrate concrete pores and chemically bond with the surface, forming a robust hydrophobic barrier [14,15]. Additionally, hybrid sol-gel coatings can be functionalized with various groups, such as alkyl, fluoroalkyl, or aromatic compounds, to tailor their hydrophobicity and durability [15,16].

Compared with fluorine-based compounds, which are effective but raise significant environmental and health concerns, sol-gel coatings offer a more sustainable alternative. While fluorine-containing coatings, such as polyfluorinated alkyl silanes (PFASs), have been shown to enhance water repellency due to their low surface energy, their environmental impact cannot be ignored. Regulatory restrictions on the use of fluorinated materials have increased the demand for eco-friendly alternatives [17,18].

Combining fatty acids with functional groups that can react with hydroxyl groups on the concrete surface involves innovative chemical strategies. Our earlier articles present the synthesis of an alkoxysilyl derivative based on rapeseed oil by nucleophilic substitution [19] and hydrosilylation [20] reactions. The obtained derivative has been used for the production of wood [19], steel [18], and concrete [20] surface coatings that protect against the adverse effect of water.

Studies have demonstrated the versatility and efficiency of the thiol-ene click reaction in various applications, such as the modification of glass [21] and textile [22,23] surfaces with hydrophobic moieties. Additionally, it has been used to modify concrete with functionalized polybutadienes [24]. In the presented applications, the derivatives obtained as a result of the thiol-ene reaction were attached to modified surfaces in the sol-gel process. The sol-gel process takes place under mild conditions, which is suitable for the modification of concrete surface modifications. The sol-gel reaction can be facilitated using base or acid catalysis, enhancing its applicability in environments requiring concrete modification. This method ensures a strong, covalent bond formation between the fatty acids and the concrete surface, thereby creating durable, hydrophobic coatings.

In this publication, we describe a method for formatting protective coatings from fatty acids using the thiol-ene click reaction and sol-gel process. The efficiency of the thiol-ene reaction was confirmed by NMR spectroscopy, showing the successful formation of the silane compound (UVMeS). Concrete samples were treated with the synthesized UVMeS solutions, both by immersion and brush applications, followed by an evaluation of their wettability and water absorption properties. The results indicated a substantial reduction in surface wettability, with water contact angles consistently above 93°, and a significant improvement in water barrier properties, reducing water permeability by more than 90%. The penetration depth of the coatings was effectively achieved, with silane solutions reaching depths of 5–7 mm for immersion and 3–5 mm for brushing applications. These findings suggest that our environmentally friendly approach provides a viable alternative to traditional fluorine-based compounds to improve concrete durability and hydrophobicity.

## 2. Materials and Methods

### 2.1. Materials

Technical grade methyl oleate (70%); 3-mercaptopropyltrimethoxysilane (95%) and 2,2′-dimethoxy-2-phenylacetophenpne (DMPA, 99%), used for the synthesis of UVMeS, were purchased from Sigma-Aldrich. Tetraethoxysilane (TEOS, 98%) was also purchased from Sigma-Aldrich (Poznań, Poland). Hydrochloric acid (35–38%) and ethanol (96%) were supplied by Avantor Performance Materials Poland S.A. (Gliwice, Poland). Demineralized water used for the preparation of sol-gel solutions and in all tests was prepared in the laboratory; only tap water was used for the preparation of concrete samples. The chemicals were used without further preparation. The concrete samples were composed of Portland type 32.5 cement (GÓRAŻDŻE CEMENT S.A., Górażdże, Poland), prepared according to EN 197-1 [25], using gravel with a maximum grain size of 16 mm and sand with a maximum grain size of 4 mm (Kruszgeo, Rzeszów, Poland).

### 2.2. Synthesis of UVMeS Silane Based on Methyl Oleate-Based Silane UVMeS

**Figure 1:**
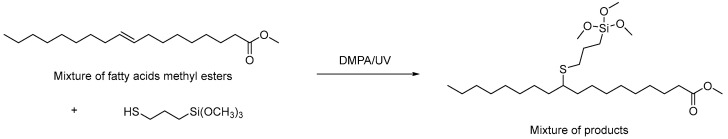
Synthesis of UVMeS via the thiol-ene reaction.

In the experiment, UVMeS silane was synthesized using commercially available methyl oleate (technical grade, 70%, Sigma-Aldrich). The UVMeS synthesis is shown below (Figure 1.).

DMPA (1.7 g, 0.00674 mol) was added to the mixture of methyl oleate (100.0 g, 0.337 mol) with 3-mercaptopropyltrimethoxysilane (66.2 g, 0.337 mol). The mixture was stirred and irradiated for 1 h (medium pressure mercury lamp 400 W; 280–600 nm) at room temperature. The reaction was carried out in air in a laboratory UV reactor from Heraeus (Pittsburgh, PA, USA). The UV lamp was placed in a water cooler to limit heating of the reaction mixture by the operating UV lamp. After completion of UV irradiation, the excess of thiol silane was then removed by reduced pressure.

NMR analyses of the obtained mixture confirm the complete conversion of the -C=C- double bonds. NMR spectra are included in the Supplementary Materials.

^1^H NMR (C_6_D_6_, 298 K, 500 MHz) δ = 0.62 (t, 2H, SiCH_2_-); 0.85 (t, 3H, -CH_3_); 1.24–1.59 (m, 30H, -CH_2_-); 1.93 (t, 2H, C(O)-CH_2_-); 2.27 (t, 2H, -S-CH_2_-); 2.52 (m, 1H, -S-CH-); 3.44 (s, 3H, CH_3_-O-C(O)-); 3.54 (s, 9H, Si-O-CH_3_).

^13^C NMR (C_6_D_6_, 298 K, 126 MHz) δ = 8.16 (SiCH_2_-); 14.04 (-CH_3_); 22.63–34.02 (-CH_2_-); 45.73 (-S-CH-); 50.42 CH_3_-O-C(O)-); (51.32 (Si-O-CH_3_); 174.21 (C=O) ppm.

^29^Si NMR (C_6_D_6_, 298 K, 99 MHz) δ = −42.56 ppm.

### 2.3. Concrete Preparation Procedure

Concrete specimens were prepared using a blend comprising 1295 kg/m^3^ of coarse aggregate (gravel), 595 kg/m^3^ of fine aggregate (sand), 380 kg/m^3^ of cement, and 190 kg/m^3^ of water. The water-to-cement ratio (*w*/*c*) was maintained at 0.5. The freshly mixed concrete was placed into molds (samples size: 100 × 100 × 100 mm) and vibrated. After 24 h of curing, the cubic samples were demolded and then placed in an oven under controlled conditions (20 ± 2 °C, relative humidity ≥ 90%) for a curing period of 28 days.

### 2.4. Preparation of a Hydrophobic Coating

After curing, the concrete samples were cleaned with water to remove loose particles and then dried for 5 days. Following this, the samples underwent silanization. Two series of solutions were prepared using UVMeS and tetraethoxysilane (TEOS). The first solution (U1) was created by combining 25 g of UVMeS, 5 g of water, 5 g of concentrated hydrochloric acid, and 465 g of ethanol. The second solution (U2) consisted of 25 g of UVMeS, 25 g of TEOS, 25 g of water, 5 g of concentrated hydrochloric acid, and 420 g of ethanol. These mixtures were stirred for either 3 h (U1.3 and U2.3) or 72 h (U1.72 and U2.72).

After the designated stirring time, the solutions were applied for silanization of the concrete surface. Two methods were used: immersion and brushing applications. In the immersion method, the concrete was immersed in the solutions for 1 h, followed by drying at room temperature for 24 h. In the brushing application method, solutions were applied to concrete surfaces with a brush, with repeated applications after 2 h. Subsequently, the concrete samples were then allowed to dry at room temperature for an additional 24 h.

The consumption of the silane solution was determined by measuring the weight loss of the UVMeS solution (in grams) per unit area of the silanized surface (in square meters). The mass loss of the UVMeS solution was calculated based on the mass differences of the silanizing solutions before and after placing the samples in them (for the immersion method) or before and after painting (for brush painting). Additionally, when painting with a brush, the solution was carefully squeezed out of the brush. The surface area of the concrete samples was calculated based on their shape without taking into account the porosity of the material. Then, the consumption of silanizing solutions was converted into the consumption of pure UVMeS.

### 2.5. Analyses and Measurements

Scanning electron microscopy (SEM) images were taken on an FEI Quanta 250 FEG microscope equipped with an EDAX energy-dispersive spectroscopy detector (EDS). The images were taken in high-vacuum mode with a 10 kV accelerating voltage. For the EDS mapping, an electron beam energy of 20 keV was employed, utilizing the EDS Octane SDD detector (EDAX). SEM images were captured of concrete samples that were cut into 10 × 10 × 10 mm cubes and that had undergone silanization. To prepare the concrete samples for SEM imaging, they were attached to standard SEM carbon adhesive tape.

Static water contact angle (WCA) measurements for all samples were carried out using a Krüss GmbH (Hamburg, Germany) DSA 100 expert drop shape analyzer equipped with software controlled (DAS4 2.0): x-, y-, and z-axis tables; quadruple dosing unit with zoom and focus adjustment; illumination; and a camera with a resolution of 780 × 580 pixels. All data presented represent the arithmetic means of measurements taken from 5 drops per sample. Contact angle measurements were performed immediately after the droplet was deposited onto the studied surface.

In an additional experiment to assess the hydrophobic character of the concrete surface, a water droplet containing 0.1% methyl orange solution with a volume of 20 µL was deposited on the surface of the concrete sample.

To determine the water absorption of concrete cubes, a liquid water permeability test was conducted in accordance with the EN 1062-3 standard [26]. Before testing, the concrete samples underwent conditioning involving three cycles of drying and immersion, each lasting 24 h. The samples were initially weighed to the nearest 0.1 g before immersion in water. The water level during the testing was maintained at 5–10 mm above the top surface of the concrete sample. Each test sample was placed on a plastic rack with 10 mm clearance above the base of the container. Any air bubbles adhering to the concrete surface were carefully removed by wiping with a clean, damp, lint-free cloth 10 min after the test commenced. After 24 h, the test sample was removed from the water, dried with absorbent paper, and weighed again to the nearest 0.1 g. The relative liquid water during the 24 h period (*w*) was calculated using the following Equation (1):

(1) $$w=\frac{\Delta m}{\surd 24\times A}\;[kg/(m^{2}\times h^{0.5})]$$

where Δ*m* is the mass variation before and after submersion (kg); *A* is the surface area of the sample (m^2^).

The penetration depth of the silanization solution was evaluated according to the EN 1504-2 standard [27]. After silanization, concrete cubes were fractured into two parts. The fractured surfaces were then sprayed with water. The boundary between the bright area (silanized region not wetted with water) and the dark area (region wetted by water) indicates the depth of penetration of the silanization solution into the concrete. This method provides a visual indication of how deeply silane penetrated the concrete substrate.

## 3. Results and Discussion

Functionalized/Silylated methyl oleate was obtained via thiol-ene click reactions. The reaction using sulfur-containing derivatives is an excellent alternative to the hydrosilylation reaction, which does not occur for internal double C-C bonds. The reaction was carried out in the presence of 2,2-dimethoxy-2-phenylacetophenone (DMPA) as the photoinitiator. 2,2-azobis(2-methylpropionitrile) (AIBN) and the benzophenone/UV system were also tested, but only partial substitution of multiple bonds was obtained, not exceeding 60%. However, when DMPA was used as a photoinitiator, almost complete conversion of double bonds was achieved (based on ^1^H NMR). It should be emphasized that the methyl oleate used is a technically pure product containing approximately 70% of methyl oleate. The profile of higher fatty acid esters corresponds to olive oil [28]. The mixture also contains polyunsaturated fatty acids (about 10%) and saturated fatty acids (15%). The amount of 3-mercaptopropyltrimethoxysilane allows the addition to all double bonds. The methyl oleate used for synthesis was obtained from vegetable oils, and due to its natural origin, it is a mixture of methyl esters of various fatty acids. The technical grade mixture (methyl oleate) was mainly used for economic reasons. The isolation or use of commercially available pure methyl oleate would significantly increase the costs of obtaining UVMeS.

As discussed in the experimental section, before silanizing the concrete surface with the solutions, they were stirred for 3 and 72 h. These stirring times were selected based on previous experiments, visual observation, and ^29^Si NMR analyses (unpublished data). After adding silanes to acidified alcohol, a hydrolysis reaction began to occur, and the highest silanol concentration was observed after 3 h. Silanols are more soluble than the initial alkoxysilane, and we observe the solution becoming clear (the initial solution after adding silanes was opalescent). After about 72 h, the solution was observed to become cloudy again, and partial condensation of silanols occurred. Due to the low concentration of alkoxysilanes in the initial solutions, the condensation process is slow and small. Only after applying silanizing solutions to the concrete surface in the form of a thin layer does the solvent evaporate quickly and the silanol concentration increase. In these conditions, the Si-OH groups undergo condensation. UVMeS infiltrates the concrete pores and interacts with their surfaces through a sol-gel mechanism, resulting in the formation of Ca/Si-O-Si bonds (refer to [20] for example). Chemically, UVMeS (see Figure 1) is an ester of higher fatty acids and can decompose on the concrete surface to produce a calcium oleate derivative, analogous to the use of butyl stearate as a concrete additive [29]. This compound, when used in fresh concrete, slowly reacts to form water-insoluble calcium stearate. For the silanization of the concrete surface, the influence of this process is expected to be minimal.

In this study, two methods for applying protective coatings were utilized: immersion of the concrete samples in a silane solution and brushing the silane onto the concrete surface twice. The immersion method is commonly used in academic research but is impractical to silanize large concrete structures in real-world applications. Both coating application methods showed similar UVMeS consumption. On average, the UVMeS consumption was 20.3 ± 1.6 g/m^2^ for the immersion method and 21.5 ± 1.9 g/m^2^ for the double brushing method.

**Figure 2:**
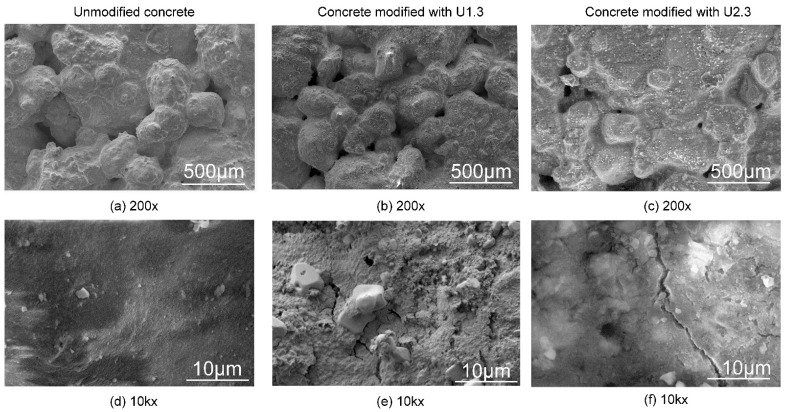
SEM images of unmodified concrete (**a**,**d**) and concrete with a modified surface, samples U1.3 (**b**,**e**) and U2.3 (**c**,**f**).

The description of the SEM images provides an overview of the effects of silanization on concrete surfaces. Figure 2 displays representative SEM images of concrete surfaces before and after silanization. The raw concrete (Figure 2a) surface appears rough but uniformly so, with noticeable porosity. In post-silanization SEM images (Figure 2b,c), an additional layer is evident, covering the previously porous surface. Despite this, silanization does not completely fill the concrete’s pores, as open pores remain visible at 200× magnification. This suggests that the silanization process, while adding a hydrophobic layer, does not obstruct the porous nature of the concrete. Figure 2e,f, taken at a higher magnification (10k×), show characteristic cracks and fissures in the layer covering the concrete surface, typical of silica structures produced via the sol-gel method. Notably, the surface treated with solutions containing TEOS (Figure 2f) shows structures resembling amorphous silica layers. The SEM images confirm that UVMeS functions as a typical hydrophobic impregnation agent, enhancing the concrete’s water resistance without blocking its porous structure.

**Figure 3:**
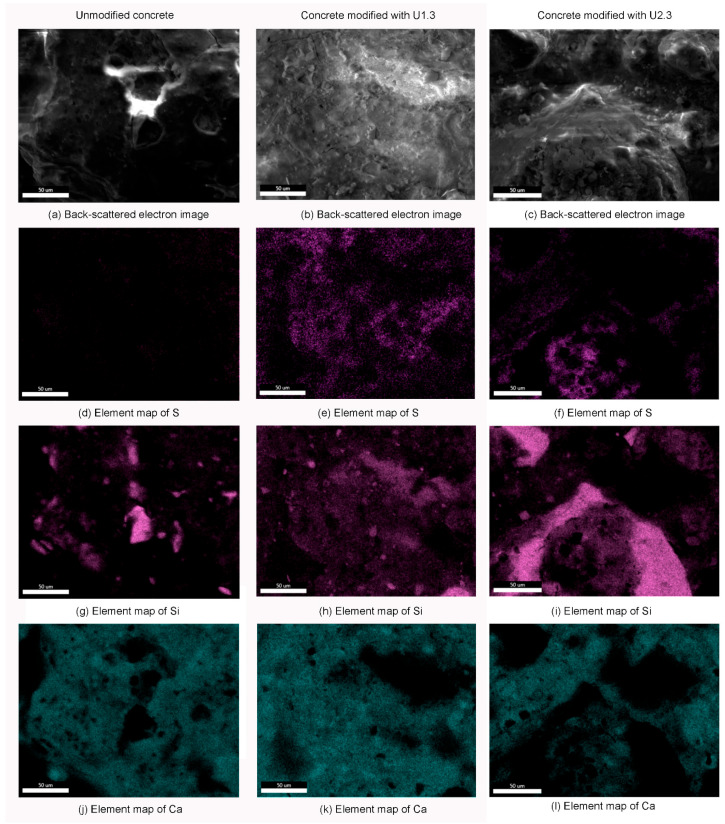
EDS mapping of elements that occur on the surface of unmodified concrete and silanized samples U1.3 and U2.3.

Figure 3 presents the EDS (energy-dispersive spectroscopy) mappings of sulfur, silicon, and calcium atoms on concrete surfaces, highlighting the elemental distribution before and after silanization. The EDS mappings of unmodified concrete reveal predominant silicon and calcium, with silicon being more concentrated in areas with less calcium. This distribution reflects the inherent composition of the concrete mixture. The sample U1.3 shows a high sulfur content due to the presence of UVMeS. The sulfur distribution correlates well with the silicon distribution, indicating that the siloxane layer from UVMeS covers the concrete surface relatively uniformly. The sample U2.3, which includes additional TEOS, demonstrates a different pattern. Here, silicon-rich regions are observed without corresponding sulfur, suggesting that areas covered by TEOS are visible. Additionally, the presence of cracks in the TEOS layer indicates issues with the uniformity of the coating, potentially caused by the TEOS addition. In summary, the EDS mappings confirm that UVMeS contributes to a more uniform coverage of the concrete surface, while the addition of TEOS results in a less consistent coating, evident from both the increased silicon content and the appearance of cracks.

The WCA measurements are summarized in Table 1.

The contact angle measurements provide insights into the hydrophobic properties of the concrete surfaces before and after treatment. Accurate measurement of the contact angle for raw concrete was not possible because water droplets immediately spread and were absorbed into the surface. This behavior indicates a high level of water absorption and a strongly hydrophilic nature of the unmodified concrete. For concrete surfaces modified with UVMeS alcohol solutions, the average contact angles were measured at above 93°. This result confirms the successful imparting of hydrophobic properties to the concrete surfaces. The contact angles for all modified samples were consistent, with only minor variations observed. Concrete surfaces treated with UVMeS using a brushing application technique exhibited slightly lower contact angles compared with other modification methods. Despite this difference, the values remained within the range of standard deviations, indicating that the variation in contact angles was not statistically significant. In summary, the contact angle measurements affirm that UVMeS effectively renders concrete surfaces hydrophobic, with minor variations in contact angle values based on the application method. It should be additionally emphasized that the samples with the addition of TEOS have a tendency to slightly spread (larger deviations and visual observations); for some drops, a reduction in the angles was observed over time (e.g., 89 by 82; 102 by 93, respectively). In general, the surface of the samples with the addition of TEOS is diversified. Comparing the obtained results with our previous works, we observe a decrease in the hydrophobic nature of the modified surface. By modifying the concrete surface with octyltriethoxysilane, a wetting angle of 125° was obtained. In the case of modification with an alkoxysilane with a fatty acid attached via a carbonyl group, this angle was approximately 115° [20]. In the case of UVMeS, the hydrocarbon chain forms a more branched system; the carboxyl group is located “at the end” of the molecule and may affect the decrease in the hydrophobic nature of the modified surface, for example by an easier formation of hydrogen bonds.

**Figure 4:**
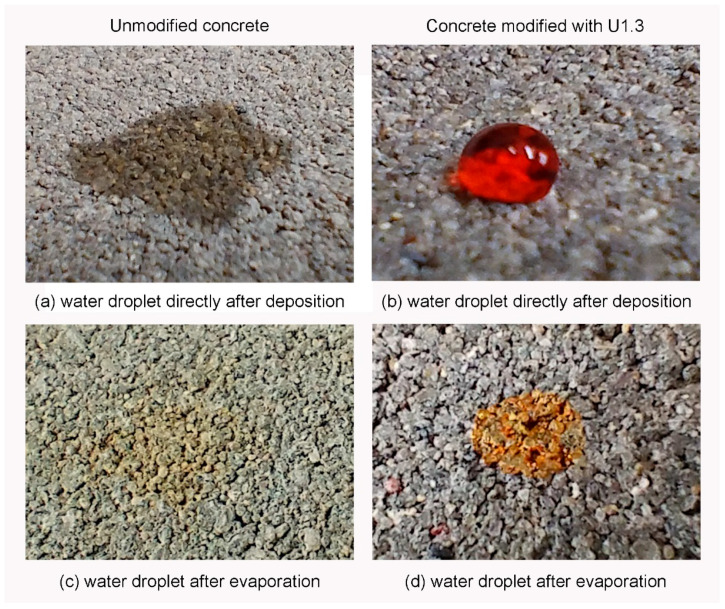
Images of a water droplet dyed with methyl orange placed on the surface of the concrete directly after deposition and after evaporation.

Figure 4 illustrates the behavior of water droplets dyed with methyl orange on both unmodified and silanized concrete surfaces, providing visual evidence of the effects of surface modification. The water droplet spreads quickly over a large, irregular area and is absorbed into the unmodified concrete surface, demonstrating the high water absorption and hydrophilic nature of the unmodified concrete. After evaporation (Figure 4c), the dye residue left behind is irregularly shaped and smaller than the initial droplet, indicating significant capillary action and absorption into the surface. The water droplet retains a more regular shape with a minimal contact area with the surface, reflecting the hydrophobic nature of the silanized concrete (Figure 4b). After evaporation (Figure 4d), the dye stain remains in a regular, circular shape, corresponding closely to the initial droplet’s size. This result indicates that the water did not spread significantly and was repelled by the hydrophobic surface. These observations are consistent with the contact angle measurements previously discussed. They confirm that silanization effectively imparts hydrophobic properties to the concrete, as evidenced by the behavior of water droplets on the treated surfaces. All modified concrete samples showed similar behavior, reinforcing the uniformity of the silanization effect across different samples.

As mentioned above, the absorbability measurements were performed according to EN 1062-3 [20]. The water absorbability (*w*) data are summarized in Table 2:

The water absorption results reveal significant insights into the effectiveness of silanization treatments for concrete surfaces. For unmodified concrete, the water absorption levels were consistent with those reported by Baltazar et al. [30], indicating the high permeability typical of untreated concrete. In contrast, silanized concrete samples showed a marked improvement in water resistance. Specifically, for samples U1.3 and U1.72, which were treated by immersion in UVMeS-containing solutions, water absorbability decreased by 91.1% and 89.9%, respectively. This substantial reduction highlights the enhanced water resistance imparted by the UVMeS treatment. However, it was noted that prolonged stirring of the UVMeS solution slightly diminished the coating’s barrier effectiveness. The addition of TEOS further improved the results. Concrete samples U2.3 and U2.72, which were treated with solutions containing TEOS, exhibited even greater reductions in water absorbability, with decreases of 92.3% and 93.6%, respectively. This indicates that TEOS enhances the hydrophobic properties of the coating. Interestingly, despite the presence of heterogeneous layers, cracks, and fissures observed in SEM and EDS analyses, these imperfections did not significantly compromise the coating’s ability to prevent water penetration. When it comes to the application method, brush-applied coatings generally showed minor increases in water absorbability. Nonetheless, sample U2.72, which was treated with TEOS and applied by brushing, achieved a notable 94% reduction in water absorbability, suggesting that the method of application can influence the coating’s performance, particularly when using TEOS-containing solutions. Overall, these results underscore that the addition of TEOS enhances the barrier properties of silanized coatings, with the coatings maintaining their effectiveness even when imperfections are present. The impact of longer stirring times appears minimal for solutions containing TEOS.

Additionally, the obtained results were also compared with coatings obtained on the basis of the commercially available Silicone L6. Coatings based on the Silicone L6 were prepared according to the manufacturer’s instructions by painting the concrete surface twice with a brush. These results are collected in Table 3. Samples protected with Silicone L6 showed a relative water permeability of 0.0964, at least twice as high as those obtained for coatings based on UVMeS.

**Figure 5:**
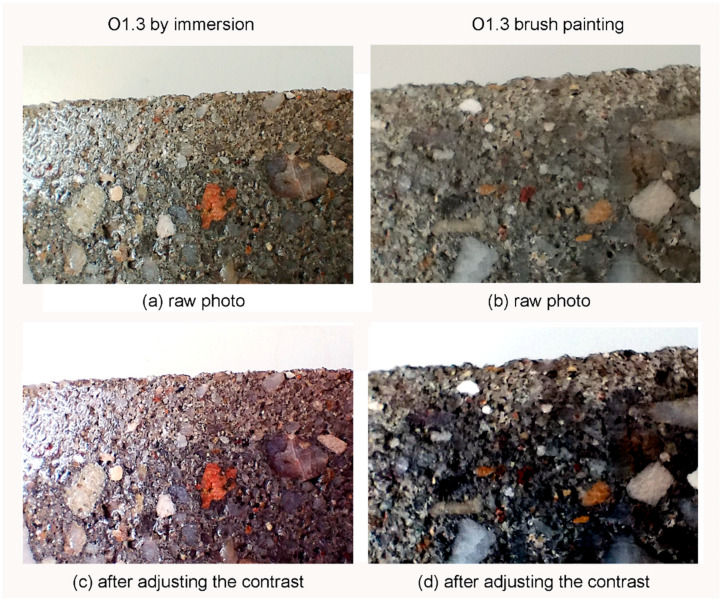
Results of revealing the penetration depth by water spraying.

The depth of silane penetration into the concrete was evaluated according to the EN 1504-2 [27] standards. Concrete cubes were cut, and the crack surfaces were sprayed with water to assess the penetration. Figure 5 provides examples of these wet cutting surfaces, where the silane-coated areas remained noticeably unwetted and appeared significantly brighter compared with the deeper layers of the concrete, which were thoroughly wetted. For concrete samples modified by immersion in silane solutions, the penetration depth of the silane reached between 5 and 7 mm. This indicates a substantial depth of treatment. In contrast, surfaces that were treated with a brushing application exhibited slightly shallower penetration depths, ranging from 3 to 5 mm. This difference suggests that immersion techniques generally result in deeper silane penetration compared with brushing application methods.

## 4. Conclusions

This manuscript presents advancements in the development of protective coatings for concrete, emphasizing environmentally friendly alternatives to fluorine-based hydrophobic treatments.

- Our research demonstrates that UVMeS effectively functionalizes concrete surfaces, providing a significant hydrophobic effect, as indicated by the water contact angle measurements.
- Both the immersion and brushing application methods for UVMeS were effective, with similar material consumption. However, the immersion method showed slightly better performance in reducing water permeability, with the highest reduction of water absorbability being observed in samples treated with UVMeS solutions containing TEOS.
- The inclusion of TEOS in the UVMeS solution resulted in better barrier properties against water absorption, as evidenced by reduced water permeability. However, the heterogeneity introduced by TEOS in the coating layers did not negatively impact the overall protective performance, despite some observed surface cracks.
- SEM and EDS analyses confirmed that UVMeS forms a consistent layer on the concrete surface. Higher magnifications revealed cracks typical of silica structures, with TEOS inclusion, which correlates with the complex and potentially less uniform surface morphology.
- UVMeS-based coatings outperformed the commercially available Silicone L6 in terms of water permeability, indicating that UVMeS provides a more effective hydrophobic barrier for concrete protection.
- The results suggest that UVMeS can be a viable and cost-effective alternative for concrete surface protection.

In conclusion, the innovations discussed in this manuscript not only advance concrete technology but also contribute to the broader goals of sustainability and environmental protection in construction. Continued exploration and optimization of these materials will be essential in meeting the evolving demands of the construction industry and ensuring long-term structural performance.

## Figures and Tables

**Table 1:** Water contact angle (WCA) values of concrete without and with UVMeS treatment.

Sample	WCA/Degree
Concrete.	-
Immersion	
U1.3	95.6 ± 3.71
U1.72	93.1 ± 2.99
U2.3	96.2 ± 7.46
U2.72	97.2 ± 8.01
Brush painting	
U1.3	98.4 ± 2.62
U1.72	93.9 ± 1.80
U2.3	95.8 ± 7.41
U2.72	97.8 ± 4.78

**Table 2:** Water permeability (*w*) after 24 h of immersion of concrete samples without and with UVMeS treatment.

Sample	*w* [kg/m^2^h^0.5^]	Relative Improvement in Absorbability [%]
Concrete	0.4777 ± 0.0420	-
Immersion (20.3 ± 1.6 g/m^2^)		
U1.3	0.0424 ± 0.0027	91.1
U1.72	0.0484 ± 0.0032	89.9
U2.3	0.0367 ± 0.0037	92.3
U2.72	0.0306 ± 0.0012	93.6
Brush painting (21.5 ± 1.9 g/m^2^)		
U1.3	0.0481 ± 0.0033	89.9
U1.72	0.0485 ± 0.0032	89.8
U2.3	0.0410 ± 0.0029	91.4
U2.72	0.0287 ± 0.0021	94.0

**Table 3:** Relative water permeability (*w*) after 24 h of immersion of concrete samples without and with silane treatment.

Sample	*w* [kg/m^2^h^0.5^]
Concrete	0.4777
Silicone L6	0.0964
U1.3	0.0424

## Data Availability

The original contributions presented in the study are included in the article, further inquiries can be directed to the corresponding author.

## Supplementary Materials

The following are available online at https://www.mdpi.com/article/10.3390/ma17174432/s1, Figure S1: ^1^H NMR spectra of UVMeS/DMPA, Figure S2: ^13^C NMR spectra of UVMeS/DMPA, Figure S3: ^29^Si NMR spectra of UVMeS/DMPA.
