# Supplementary material for: Protective Coatings Based on the Organosilicon Derivatives of Fatty Acids Obtained by the Thiol-Ene Click Reaction
Source: Materials (Basel). 2024 Sep 9;17(17):4432. doi: 10.3390/ma17174432 (PMC11396091; doi:10.3390/ma17174432)
Supplement: Supplementary file 1 [file materials-17-04432-s001.zip › materials-3137651-supplementary.pdf]

Protective Coatings Based on the Organosilicon Derivatives of Fatty Acids Obtained by the Thiol-Ene Click Reaction

Supporting Information

Karol Szubert, Albert Liberski

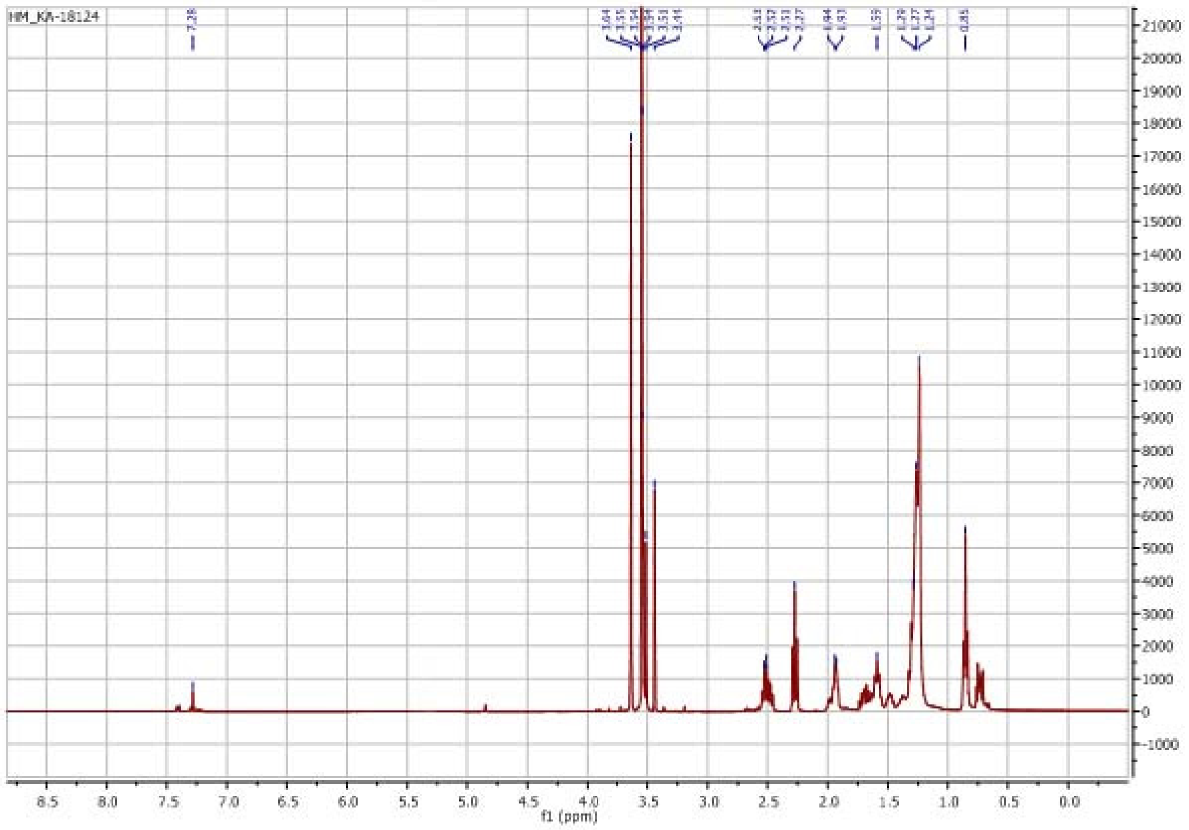

Figure S1: ^{1}H NMR spectra of UVMeS/DMPA

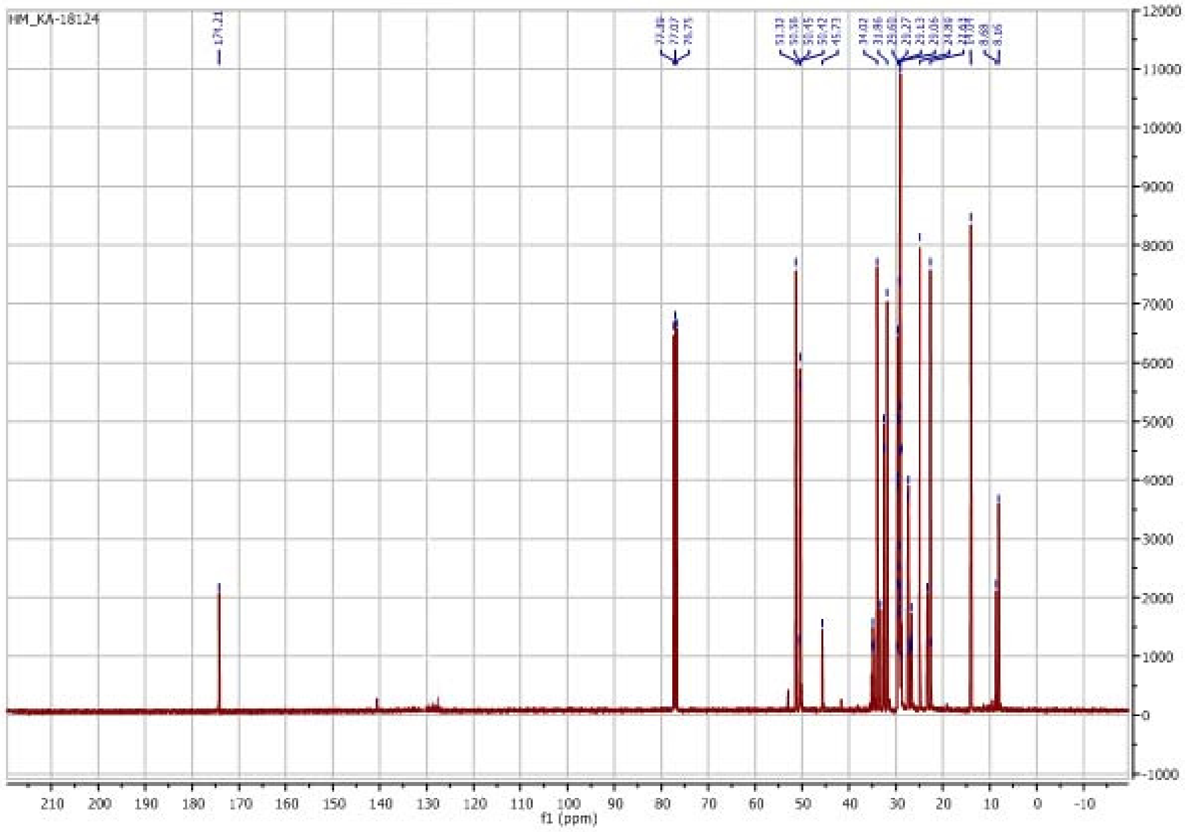

Figure S2: ^{13}C NMR spectra of UVMeS/DMPA

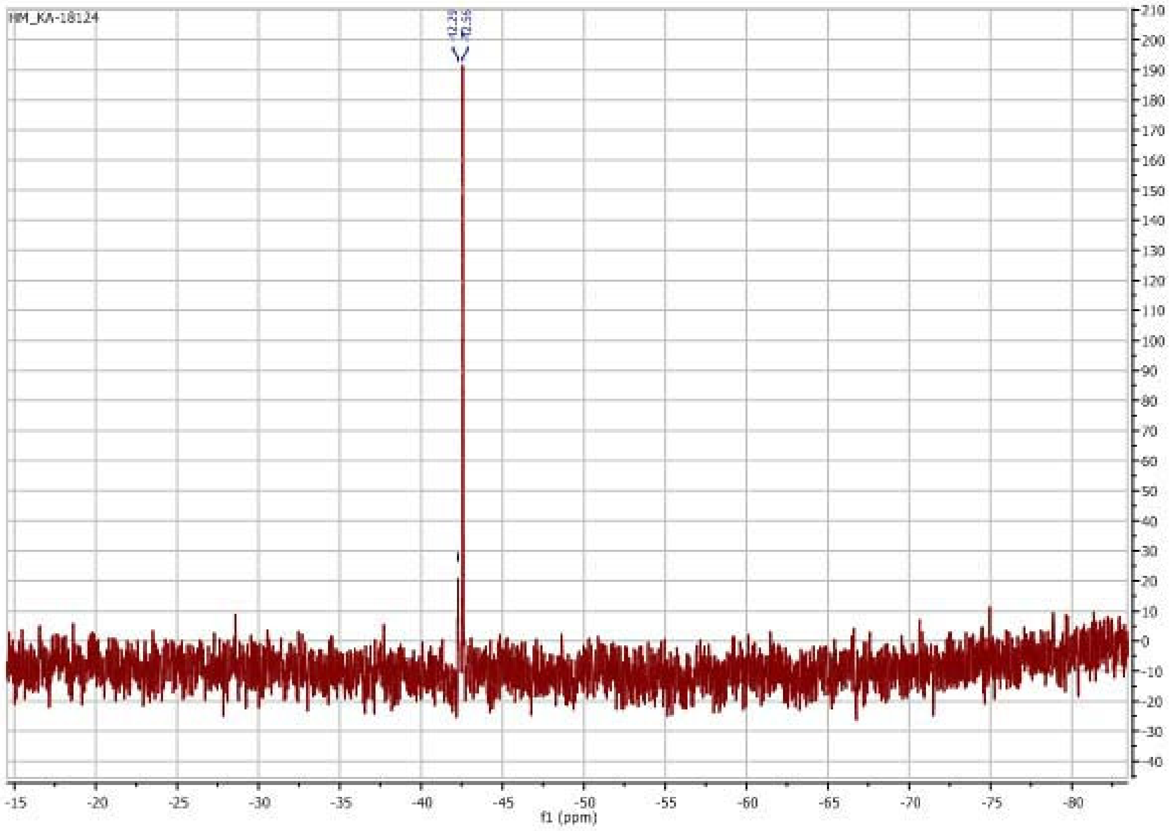

Figure S3: ^{29}Si NMR spectra of UVMeS/DMPA
